# Fluxome analysis using GC-MS

**DOI:** 10.1186/1475-2859-6-6

**Published:** 2007-02-07

**Authors:** Christoph Wittmann

**Affiliations:** 1 Biochemical Engineering Institute, Saarland University, Saarbrücken, Germany

## Abstract

Fluxome analysis aims at the quantitative analysis of in vivo carbon fluxes in metabolic networks, i. e. intracellular activities of enzymes and pathways. It allows investigating the effects of genetic or environmental modifications and thus precisely provides a global perspective on the integrated genetic and metabolic regulation within the intact metabolic network. The experimental and computational approaches developed in this area have revealed fascinating insights into metabolic properties of various biological systems. Most of the comprehensive approaches for metabolic flux studies today involve isotopic tracer studies and GC-MS for measurement of the labeling pattern of metabolites. Initially developed and applied mainly in the field of biomedicine these GC-MS based metabolic flux approaches have been substantially extended and optimized during recent years and today display a key technology in metabolic physiology and biotechnology.

## Background

The present review describes the use of stable isotopes, mainly ^13^C, for the analysis of metabolic fluxes. In all the approaches presented here, single or multiple labeled molecules, i.e. applied tracer substrates or analyzed metabolites, play an important role. Concerning the nomenclature of such molecules labeled with stable isotopes there are no strict IUPAC guidelines. Labeled molecules are identified by the number and position(s) of heavy isotopes in their constitutive atoms. Most commonly the following definitions and terms are used by researchers in the field of metabolic flux analysis. They are exemplified for the carbon isotopes ^12^C and ^13^C, but can be generally applied for other isotope labels as well. *Positional isotopomers *have an exactly determined labeling pattern with a specific number of ^13^C atoms in specific positions of the molecule. Different positional isotopomers thus exhibit identical global isotopic composition but differ by the position of the heavy atoms in the molecule. For example, [1-^13^C] pyruvate and [2-^13^C] pyruvate are positional isotopomers with the ^13^C label at carbon positions C_1 _and C_2_, respectively. Generally, 2^n ^positional isotopomers are possible for a compound with n carbons. *Mass isotopomers *differ only by the number of heavy atoms in their molecules, resulting in different molecular weights. However, they do not differ by the position of the label. For example, [^12^C_3_] pyruvate, [^13^C-^12^C_2_] pyruvate, [^13^C_2 _^12^C] pyruvate and [^13^C_3_] pyruvate are different mass isotopomers. Usually, the lightest atoms are not specified and these mass isotopomers are identified as pyruvate, [^13^C] pyruvate, [^13^C_2_] pyruvate and [^13^C_3_] pyruvate, in agreement with the International Union of Chemistry. In mass spectrometric jargon, the corresponding different mass isotopomer fractions are referred to as M_0_, M_1_, M_2 _and M_3 _or as *m*, *m+1*, *m+2 *and *m+3*. When only one element of a compound with *n *such atoms of this element is labeled, the number of possible mass isotopomers is (*n+1*). Most mass isotopomers, except m and *m+n*, include multiple positional isotopomers. It should be noted that the number of positional isotopomers of symmetrical molecules such as succinate is less than 2*n*. However, chemically symmetrical molecules may be biologically asymmetrical because of the configuration of the active site of enzymes (e.g. the citrate synthase) or metabolic channeling [1]. The ^13^C labelling state of a molecule with *n *carbons can be also expressed as molar enrichment (ME) [2] or as summed fractional labelling (SFL) [3] describing the weighted sum of mass isotopomer fractions (Equation 1).

ME=∑i=1ni⋅xm+i     (1)
 MathType@MTEF@5@5@+=feaafiart1ev1aaatCvAUfKttLearuWrP9MDH5MBPbIqV92AaeXatLxBI9gBaebbnrfifHhDYfgasaacH8akY=wiFfYdH8Gipec8Eeeu0xXdbba9frFj0=OqFfea0dXdd9vqai=hGuQ8kuc9pgc9s8qqaq=dirpe0xb9q8qiLsFr0=vr0=vr0dc8meaabaqaciaacaGaaeqabaqabeGadaaakeaacqqGnbqtcqqGfbqrcqGH9aqpdaaeWbqaaiabdMgaPjabgwSixlabdIha4naaBaaaleaacqWGTbqBcqGHRaWkcqWGPbqAaeqaaaqaaiabdMgaPjabg2da9iabigdaXaqaaiabd6gaUbqdcqGHris5aOGaaCzcaiaaxMaadaqadaqaaiabigdaXaGaayjkaiaawMcaaaaa@4382@

## Introduction

Fluxome analysis aims at the quantitative analysis of *in vivo *carbon fluxes in metabolic networks. The experimental and computational approaches developed in this area have revealed fascinating insights into various biological systems. In addition they offered new possibilities as a rational basis for targeted strain improvement [4,5]. Fluxome, i. e. metabolic flux analysis, therefore is in the core of metabolic engineering [6]. The most wide spread approaches for fluxome analysis are based on GC-MS measurement of labelling pattern of metabolites from the tracer studies performed. Initially developed and applied mainly in the field of biomedicine [7-10], these GC-MS based metabolic flux approaches have been substantially extended and optimized and emerged as a key technology in metabolic physiology and biotechnology [6,11-15].

**Figure 1:**
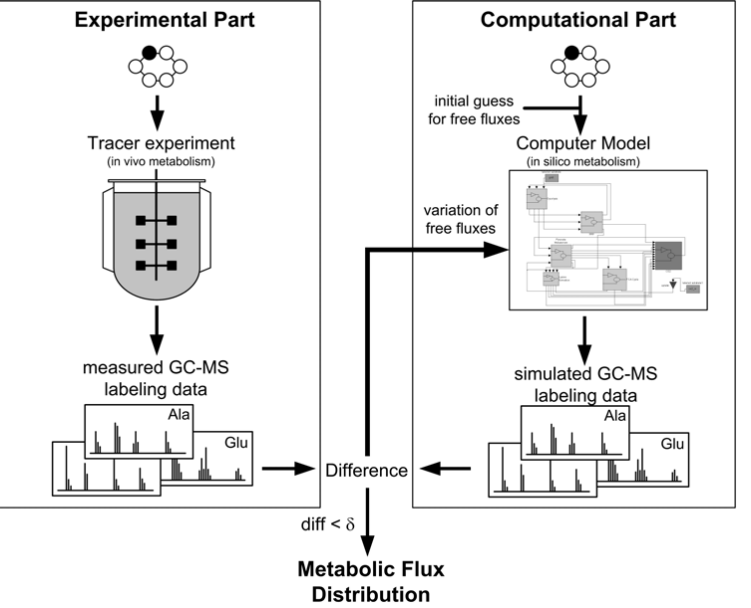
Strategy for ^13^C metabolic flux analysis including the experimental part with the tracer study and the GC-MS labelling analysis and the computational part with the simulation of the labelling data via an isotopomer model representing the investigated metabolic network. The flux estimation is based on minimizing the deviation (δ) between the measured and the simulated labelling data.

State-of art metabolic flux analysis is carried out by a comprehensive approach consisting of an experimental and a computational part (Figure 1). The organism of interest is hereby cultivated on a substrate, specifically labelled on certain positions with a stable isotope, mainly ^13^C. The labelling patterns of metabolites formed during cultivation are then measured, whereby, as stated above, GC-MS is the most used technique today. GC-MS can resolve single *mass isotopomers *of a compound differing by the number of labelled atoms and thus allows the measurement of mass isotopomer distributions. The mass isotopomer distribution of a compound can be obtained from the analysis of an ion cluster, which contains the intact carbon skeleton of the analyte. Similarly mass isotopomer distributions can be determined for certain parts of an analyte considering appropriate fragment ions containing only parts of its carbon skeleton. The mass isotopomer distribution can be used to calculate the average ^13^C enrichment of a molecule, called *molar enrichment *[2] or *summed fractional labelling *[16], via the weighted sum of mass isotopomer fractions. In selected cases, where a higher degree of labelling information is required GC-MS can be also applied to determine *positional isotopomers *[16,17]. Like a fingerprint the GC-MS labelling data sensitively reflect the intracellular fluxes and thus can be used for their calculation. For flux calculation the different labelling data obtained are usually utilized to globally fit the unknown flux parameters by a computer flux model combining isotopomer and metabolite balancing [18-22]. For a given case the flux model contains all relevant reactions and pathways of the investigated metabolic network with full information on carbon transition in each of the reactions. Most suitable for the creation of such a flux model are modelling frameworks with a systematic and general approach for the quantitative description of the transfer of labelled ^13^C atoms in metabolic networks [14,15]. Starting with a random initial guess for the free fluxes, the model computes the remaining dependent fluxes via stoichiometric mass balances and subsequently calculates the ^13^C labelling patterns of all compounds in the network for the given fluxes and compares them with the experimental labelling data. Applying an optimization algorithm the deviation of the labelling data between simulation and experiment is minimized by iterative variation of the free fluxes until the optimum fit is obtained. In combination with experimentally determined extracellular fluxes, absolute carbon fluxes throughout the network are obtained. With certain constraints GC-MS labelling data can also be used to calculate local flux ratios in the network [23,24]. Combination of these flux ratios with a stoichiometric model and measured extracellular fluxes also leads to a distribution of absolute fluxes [25]. In recent years GC-MS has been successfully applied to study fluxes in bacteria [22,23,26], yeasts [18,27,28], fungi [29], mammalian cells [30,31] or intact tissues [32,33]. The wide spread use of GC-MS for metabolic flux analysis is due to different reasons. GC-MS allows very accurate quantification of labelling patterns resulting in high accuracy and small confidence intervals of the determined fluxes. Additionally, GC-MS usually provides rich information content of the labelling data so that important fine structures of the metabolic network, e. g. fluxes through parallel, bidirectional or cyclic fluxes, can be resolved. The high separation efficiency of GC can resolve the often complex biological mixtures. Moreover GC-MS systems are easy to operate and robust. The present review provides an actual insight into experimental and computational tools for GC-MS based metabolic flux analysis, thereby focussing on the currently most important approach which involves utilization of amino acid labelling patterns. Amino acids are often measured instead of the intermediary metabolites in the central metabolism since the amino acids in cell extracts and cell protein are much more abundant than their precursors [16] and provide extensive labelling information. Knowing the precursor-amino acid relationships it is easy to deduce the labelling patterns of the precursor metabolites from labelling patterns of the amino acids. For the different steps involved in metabolic flux analysis some practical notes and tips are addressed that seem crucial for proper flux measurements.

## GC-MS instrumentation

**Figure 2:**
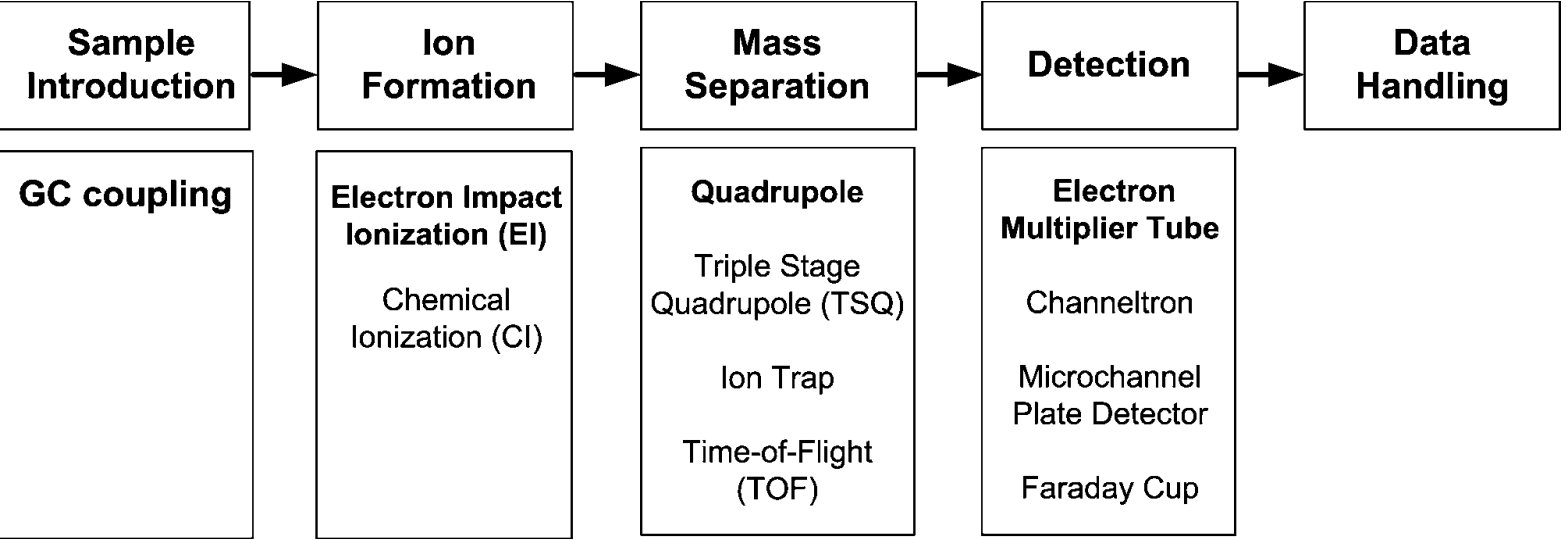
Overview on different GC-MS instrumentation types. The most frequently used combination in the area of metabolic flux studies by isotope labelling is highlighted.

GC-MS allows the measurement of various metabolites [34]. This comprises a number of volatiles such as ketones, aldehydes, alcohols, heterocyclic compounds, isocyanates, isothiocyanates, sulfides, lipids, and hydrocarbons up to 12 carbons, which all can be directly measured. Additionally, various non-volatile or semi-volatile metabolites including sugars, sugar-phosphates, sugar alcohols, organic acids, amino acids, lipids, peptides, long-chain alcohols, alkaloids, amines, amides, etc. are accessible after derivatization. For flux analysis amino acids, organic acids and sugar derivates are the most important compounds to be considered. Currently there is a wide range of instruments available varying in the type of ionization and the mass separation (Figure 2). Single-quadrupole mass spectrometers with electron impact (EI) ionization are the most often used type of instrument. Compared to other instruments they are relatively low cost and offer a range of advantages such as high robustness, high sensitivity and high accuracy of the measured labelling patterns.

### GC separation

**Figure 3:**
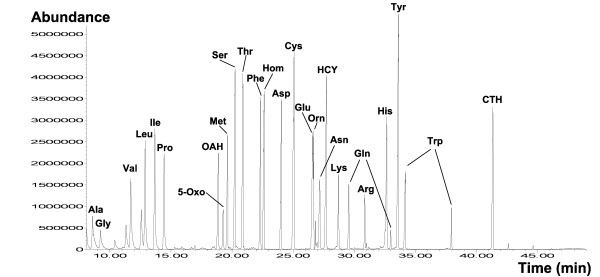
Total ion current (TIC) spectrum of a sample with TBDMS-derivatized metabolites. The separation of the totally 28 compounds is performed on a HP5-MS column (60 m, 250 μm inner diameter, Hewlett-Packard, Avondale, PA).

The sample solution (usually between 0.1 to 1 μL) is injected into the GC inlet where it is vaporized and directed onto the chromatographic column by the carrier gas (usually helium). The sample molecules are separated through different interactions between the carrier gas phase and the stationary phase. To obtain the highest resolution in GC-MS analysis, the capillary column should be carefully chosen. The best general purpose phases are dimethylsiloxane (DB-1 or equivalent) or 5 % phenyl/95%dimethlysiloxane (DB-5 or equivalent). These non-polar phases exhibit an excellent separation capability for many compounds and a generally low column bleeding. Polar columns (DB-Wax or equivalent) are useful for selected derivates such as more polar alkylated compounds. The high separation capacity of GC is illustrated in Figure 3 for a sample containing various amino acids and related compounds.

### Ionization

**Figure 4:**
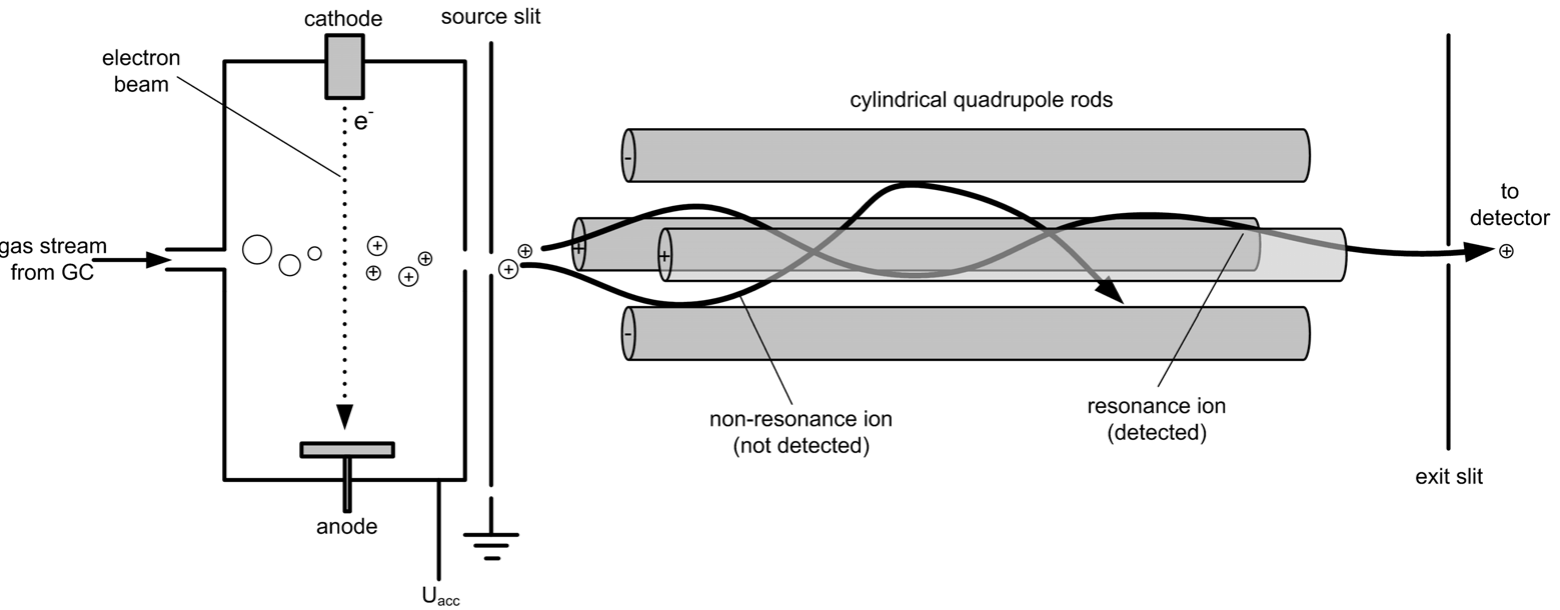
Schematic view of the ion source based on electron impact ionization and the quadrupole mass filter typically found in a GC-MS instrument.

**Figure 5:**
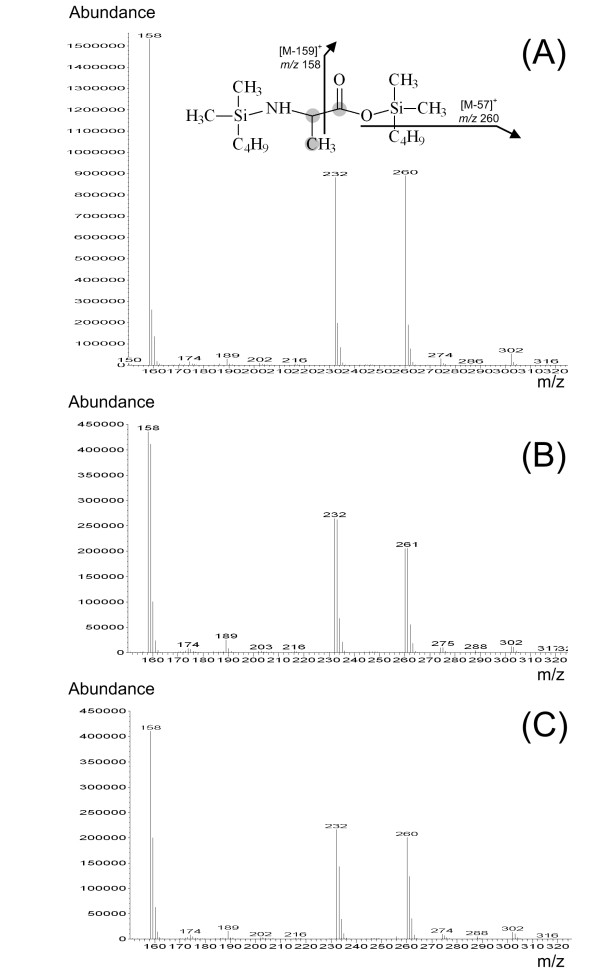
Mass spectrum of TBDMS_3_-alanine derived by electron impact ionization in GC-MS analysis: Naturally labeled alanine with a mass isotopomer distribution resulting from the nautrally occurring isotopes (A), alanine from the cell protein of *S. cerevisiae *cultivated on [1-^13^C] glucose (B), alanine from the cell protein of lysine producing *C. glutamium *cultivated on [1-^13^C] glucose (C). The monoisotopic mass of the molecular ion, which itself is not detected, is 317. The structures of valuable ion clusters for labeling analysis in metabolic flux studies (*m/z *260, *m/z *232) are additionally.

The outlet of the GC column is connected via a heated transfer line with the ion source of the MS where the compounds eluting from the column are ionized. The most frequently used ionization method is electron impact (EI) ionization (Figure 4). Hereby the interference of the sample stream with a crossing electron beam leads to the loss of an electron from the sample molecules. The resulting ions with one electron missing, i.e. the molecular ions, give the total mass of each analyte. Due to the large amount of energy imparted to the molecular ion it usually fragments, producing further smaller ions with characteristic relative abundances that provide a 'fingerprint' for that molecular structure (Figure 5A). Often the molecular ion itself is not observed. The different fragment ions may contain different parts of the carbon skeleton of the analyte. In the given example for TBDMS-derivatized alanine the ion cluster [M-57]^+^at *m/z *260 results from loss of a C_4_H_9 _group from the derivatization residue and contains all three alanine carbon atoms, whereas the ion cluster [M-85]^+ ^at *m/z *232 only contains the carbon atoms C_2 _and C_3 _of alanine due to additional loss of a CO-group including the C_1 _atom of alanine. Chemical ionization (CI) as alternative ionization method plays a minor role for flux analysis, but has proven valuable in selected cases such as labelling analysis of glucose aldonitrile pentaacetate in different medical flux studies [35-38].

### Mass separation

Through a slit the positive ions from the ion source enter into the mass separation part. Figure 3 shows the most wide spread type of mass separator in GC-MS analysis, a quadrupole, composed of four parallel cylindrical rods (about 25 cm length each), whereby opposite rods are electrically connected. The mass separation is based on the motion of ions in an oscillating electric field created through voltage variation between the rods. At a certain voltage, only analyte molecules of a distinct mass to charge (*m/z*) ratio (resonance ions) can pass and enter the detector, whereas ions exhibiting a different *m/z *ratio (non-resonance ions) are subjected to oscillations causing their collision with the rods. Within a few seconds the whole mass range available (usually 50 – 850 *m/z*) can be fully scanned. This short time interval required for a full scan is important, due to the typically narrow peaks eluting from the GC column that have to be scanned several times each (Figure 3). The sensitivity of a quadrupole detector can be significantly enhanced by selected ion monitoring (SIM), whereby only selected masses are sequentially measured with frequencies of 0.1 to 2 seconds each. The number of collected ions for the selected masses is increased up to several thousand folds in comparison to the scan mode. Quadrupole MS spectra can be easily compared with commercial or laboratory databases facilitating the identification of novel compounds and the validation that a signal is not interfered through isobaric overlay. In most cases the nominal-mass information provided by such instruments is sufficient, since the compounds analyzed are a priori known.

Other mass separators available are time-of-flight (TOF) mass separators or ion traps. In TOF instruments, ions formed in the ion source are accelerated in a short electric field to a high kinetic energy, whereby ions with lower masses reach higher velocities and need a shorter time to reach the detector. The mass to charge ratio of each ion is determined from the time elapsed from ion formation to ion arrival at the detector. This type of mass separation in GC-TOF-MS systems offers a greater mass accuracy as compared to a conventional quadrupole. This, however, is normally not required for the labelling measurement and does therefore not compensate for the relatively high price of such instruments. Only the high scan speed possible allowing multiple scanning of single peaks during their elution and shortening of analysis time [39] could be beneficial for high-throughput applications or the analysis of rather complex samples. Alternatively, ion traps can be applied for mass separation. A drawback of ion traps to be used for flux analysis is the comparatively low accuracy for the labelling measurement resulting in a higher uncertainty of the determined fluxes. Relative errors of around 1 % for the most abundant mass peaks [40] are about two to tenfold higher as compared to that of quadrupole instruments [15]. An advantage, however, is the possibility to use the ion trap as a multi stage MSn mass spectrometer which can provide more detailed information on the labelling pattern. This might be useful in cases, where a high degree of labelling information is needed to determine the fluxes of interest [41]. Similarly triple-quadrupole instruments can be run as multistage mass spectrometers. A further variant, useful e.g. for isotope labelling studies in humans or animals, is isotope ratio (IR) GC-MS [42,43]. It is based on electron impact ionization with maximized ionization probability. IR GC-MS exhibits an extremely high precision of ± 0.00001 % for the isotope ratio measurement and is optimal to quantify low label enrichment [44]. It is, however, limited to the analysis of gases of high volatility and low reactivity such as CO_2_, N_2 _or SO_2_. The analytes of interest are transformed into one of these gases before introduction into the instrument. Usually this provides information only about the specific labelling enrichment, i.e. the relative abundance of ^13^C atoms in the entire molecule. More detailed information on number and position of ^13^C can be obtained, if all carbon atoms are isolated position specific prior to measurement as recently shown for position specific ^13^C analysis of methyl palmitate through pyrolytic fragmentation [45].

## Labelling analysis with GC-MS

The analysis of the labelling pattern is a central step of metabolic flux analysis. It has to be ensured that the labelling patterns are not affected by the sampling, sample pre-treatment or the GC-MS analysis itself. The major compounds analyzed by GC-MS for flux analysis are amino acids [21,22,35,36], organic acids [35,36,46], sugars [2,37,47], lipids and fatty acids [48]. Moreover, mass distributions of polymers and their building blocks were assessed. Examples are glycogen [37,49], cell protein [16,18,24,50], or DNA[51].

### Sampling and sample pre-treatment

**Figure 6:**
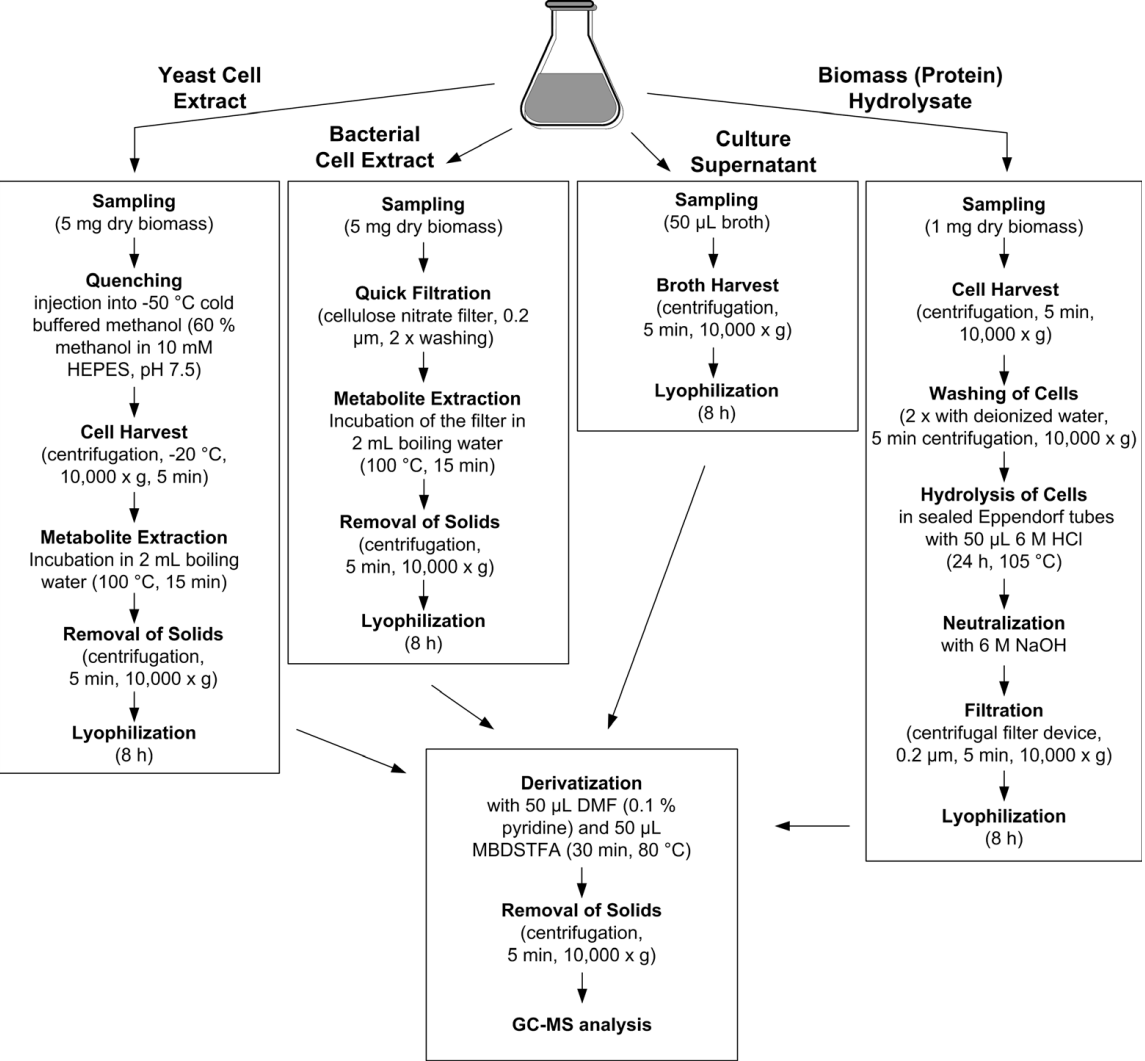
Experimental protocols for sampling and processing of amino acids in yeast and bacterial cell extracts, culture supernatant and biomass (protein) hydrolysate for GC-MS analysis.

Depending on the experiment, the analytes of interest are cellular polymers (analysis of balanced growth in chemostat or batch processes), free intracellular metabolites (analysis of process dynamics) or extracellular metabolites in the medium (analysis of production phases with reduced growth or without growth). Exemplified for amino acids, the different experimental steps of sampling and sample pre-treatment for subsequent labelling analysis by GC-MS are given in Figure 6. Free intracellular amino acids are extracted from the cells, whereby immediate quenching is important to prevent changes in the labelling pattern during sampling. Due to the high sensitivity of GC-MS only 1 mg cell dry mass are required to obtain labelling patterns from free intracellular amino acids [52]. Various quenching and fast sampling methods described in the literature for metabolome analysis can be applied here. For bacteria fast filtration with cellulose nitrate or polyamide filters (0.2 μm pore size) is suitable [53], whereas yeast cells can be efficiently quenched with cold buffered methanol [54]. The quenched cells should be washed to prevent interference with compounds contained in the medium. Metabolite extraction can be performed by incubation in boiling ethanol or in boiling water. After separation from cell debris the extract is lyophilized to concentrate the metabolites and remove the water. For the analysis of amino acids from the cell protein cells are harvested by centrifugation or filtration, washed and subsequently hydrolyzed by 24 h incubation at 110°C in 6 M HCl. During hydrolysis asparagine and glutamine are deaminated to aspartate and glutamate. Cysteine, methionine and tryptophan are destroyed through oxidation. If the hydrolysis time is shortened to about 8 h, signals can be also obtained for e.g. cysteine or methionine, but at the cost of a generally low yield. The hydrolysate is neutralized by addition of 6 M NaOH, whereby often the required volume of NaOH is less than the volume of HCl due to HCl evaporation during the hydrolysis. The pH should be checked, because the subsequent derivatization is significantly disturbed by an alkaline pH. The neutralized hydrolysate is clarified by filtration, whereby a stable glycerol-free filter material has to be chosen. Glycerol would be leached out of the filter and interfere in the later derivatization and GC-MS analysis. Finally the clarified hydrolysate is lyophilized. Extracellular metabolites are harvested by centrifugation followed directly by lyophilization.

### Derivatization

In order for a compound to be analyzed by GC-MS it must be volatile and thermally stable. Most of the metabolites utilized for flux analysis are polar or even charged and thus not sufficiently volatile to be directly analyzed, so that derivatization is typically required. This holds for amino acids [16,21,24,50], organic acids [32,35] or sugars [19,31,37,55,56]. For this purpose a number of straightforward derivatization reactions involving silylation, alkylation or acetylation are available (Table 1). The reactions are mainly simple, one-pot conversions with high yield. More details on derivatization protocols are given in recent review articles [57-62]. For the analysis of amino acids, silylation with N-methyl-N-t-butyldimethylsilyl-trifluoro-acetamide (MBDSTFA) is especially useful, since it leads to derivates which exhibit high signal intensity for the [M-57] fragment ions. These originate from loss of a *t*-butyl group from the derivatization residue, contain the entire carbon skeleton of the analyte and are thus valuable for flux calculation [63]. It should be noted that many reagents used are unstable in the presence of water, so that the aqueous samples, as described above, have to be dried and re-dissolved in organic solvent prior to derivatization. It is also possible to directly add the reagent to the lyophilized sample, using an additional solvent such as dimethylformamide, however, improves the conversion.

**Table 1:** Derivatization methods for GC-MS analysis of metabolites

**Analyte**	**Derivatization**	**Reagent**
Alcohols	Silylation	trifluoroacetamides (BSA, MSTFA, BSTFA, MBDSTFA)
Phenols	Acylation	activated carboxylates (ECF, TFAA)
	Alkylation	activated methyl groups (TMAH, TMSH, DMFDMA)
Amines (primary, secondary)	Silylation	trifluoroacetamides (BSA, MSTFA, MSHFBA)
	Acylation	activated carboxylates (TFAA, HFBA, MBTFA)
Amino acids	Silylation	trifluoroacetamides (MBDSTFA, BSA, BSTFA)
	Alkylation + Acylation	MeOH/TMCS, TMSH, DMFDMA + TFAA, HFBA, ECF, TFAA,
Carboxylic Acids	Silylation	trifluoroacetamides (BSA, MSTFA, MSHFBA, TSIM)
	Alkylation	activated methyl groups (TMAH, TMSH, DMFDMA)
a-keto acids	Oximation + Silylation	hydroxylamine, O-ethylhydroxylamine + trifluoroacetamides (BSA, MSTFA, BSTFA, MBDSTFA)
Thiols	Acylation	activated carboxylates (ECF, TFAA)
	Alkylation	activated methyl groups (TMAH, TMSH, DMFDMA)
Carbohydrates	Oximation + Silylation	hydroxylamine, O-ethylhydroxylamine + trifluoroacetamides (BSA, MSTFA, BSTFA)
	Silylation	trifluoroacetamides (BSA, MSTFA, BSTFA)
	Acylation	activated carboxylates (TFAA, MBDTFA)

### Labelling information

**Figure 7:**
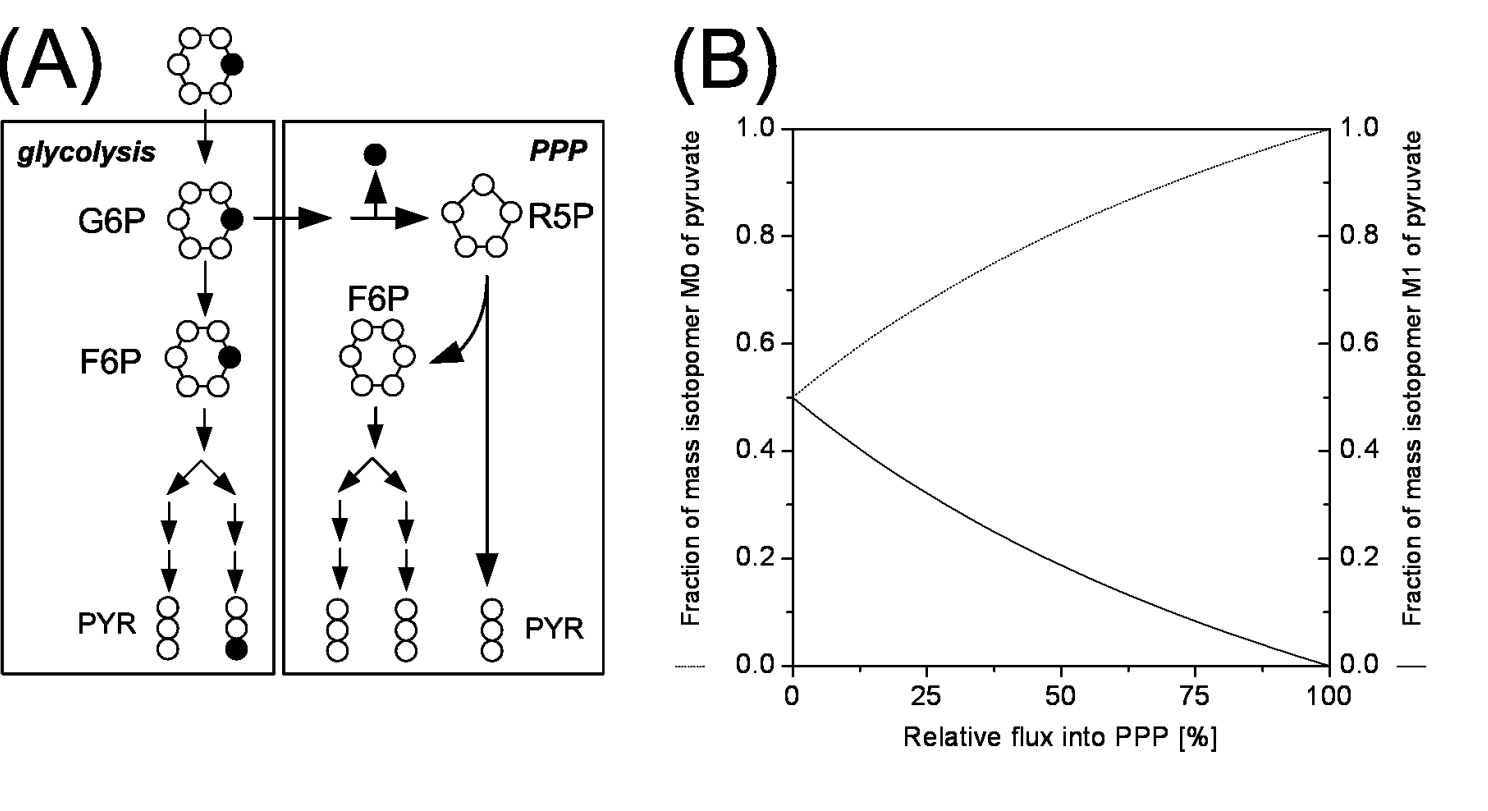
Quantification of the flux partitioning between pentose phosphate pathway (PPP) and glycolysis: Carbon transfer from [1-^13^C] glucose in the underlying metabolic reactions (A), Influence of a variation of the relative flux into the PPP on the relative abundance of non labelled (M0) and single labelled (M1) pyruvate as determined by simulation with an isotopomer model (B).

The mass isotopomer distribution of a compound can be directly taken from an ion cluster containing the entire carbon backbone of the analyte. In many cases these mass isotopomer distributions sensitively reflect the flux parameters of interest. This can be illustrated for the problem of quantifying the flux partitioning at the glucose 6-phosphate node between the pentose phosphate pathway (PPP) and glycolysis, important pathways in many microorganisms. With [1-^13^C] glucose as tracer substrate the labelled carbon atom is completely released as CO_2 _in the PPP, whereas the label is completely conserved through the different glycolytic reactions (Figure 7). The mass isotopomer distribution of pyruvate, receiving carbon from both pathways, thus significantly changes with a variation of the flux partitioning. As revealed by metabolic simulations an increase of the relative flux into the PPP is reflected by a decrease of the fraction of single labelled pyruvate and an increase of the fraction of non-labelled pyruvate. GC-MS measurement of the labelling of pyruvate or pyruvate derived metabolites such as alanine allows precise estimation of PPP and glycolytic flux. This becomes obvious from mass spectra of TBDMS-derivatized alanine from tracer experiments of a lysine producing *Corynebacterium glutamicum *mutant (Figure 5B) and *Saccharomyces cerevisiae *(Figure 4C) cultivated on 99 % [1-^13^C] glucose in batch culture. In both cases the heavier mass isotopomer fractions, e.g. at the ion cluster m/z 260, are increased due to the enrichment with ^13^C. In comparison with *S. cerevisiae *the fraction of single labelled alanine is markedly reduced in *C. glutamicum *revealing a significantly higher flux through the PPP in this organism, which is related to the increased NADPH demand for lysine production. Similarly mass isotopomer distributions of other metabolites give access to various other flux parameters, such as dual pathways in amino acid biosynthesis [64], bidirectional fluxes around the pyruvate node [64] or parallel pathways in different cellular compartments [18] and different flux ratios [24]. In other studies, summed fractional labelling data have been utilized for the analysis of fluxes [65,66]. Hereby, the lower degree of information for each single compound was compensated by the consideration of different ion fragments containing different parts of the carbon skeleton for the analytes [67]. More detailed information on the labelling of a compound can be obtained with GC-MS via additional analysis of fragment ions, which contain only specific parts of the carbon skeleton of the analyte. As example, the entire positional isotopomer distribution of pyruvate can be determined by GC-MS via analysis of four ion clusters of its methyl-ester derivate [17] or via GC-MS analysis of different ions of pyruvate derived amino acids valine and alanine [16]. More complex protocols may be required for analytes containing more carbon atoms, thus exhibiting a substantially increased number of possible positional isotopomers. The determination of 24 out of 32 positional isotopomers of glutamate involves chemical and enzymatic synthesis of five different derivates of glutamate prior to GC-MS analysis [36]. Also other protocols for resolution of positional isotopomer pools are rather laborious and include various steps for purification and chemical or enzymatic conversion of the analytes [35,49,68,69] which impedes the broad and routine use of such techniques. It should be noticed that the resolution of single positional isotopomer pools not necessarily leads to an increase in information for flux quantification [67,70], but in selected cases single isotopomer pools might be required to calculate all fluxes of interest. A major tool to obtain additional fragments towards positional isotopomer analysis is targeted fragmentation using MS/MS, also called tandem MS, where selected ions can be isolated, subsequently fragmented and the fragments can be analyzed for their mass distribution. The potential of such approaches for flux analysis is underlined by recent studies [71,72].

### Correction for natural isotopes

To calculate the ^13^C labelling of a molecule from a GC-MS measurement, the data have to be corrected for natural isotopes. Hereby, atoms of the analyte and of added derivatization residues have to be considered. Natural isotopes typically occurring in GC-MS are carbon, hydrogen, nitrogen, oxygen and silicium (Table 2). Mathematically, the presence of natural isotopes can be considered by correction matrices [17,73-75]. Straightforward is the 'addition' of the natural isotopes to the simulated data during flux calculation and the direct comparison with the measurement data [17]. Alternatively the natural isotopes can be 'subtracted' from the measurement signals to obtain the labelling of the carbon skeleton of the analyte and compare this value with the corresponding simulated data during flux calculation [76].

**Table 2:** Isotopic compositions of biologically relevant elements [129].

**Element**	**Mono isotopic mass (M)**	**Relative fraction M+0**	**Relative fraction M+1**	**Relative fraction M+2**	**Relative fraction M+4**
H	1	0.999885	0.000115		
C	12	0.9893	0.0107		
N	14	0.99632	0.00368		
O	16	0.99757	0.00038	0.00205	
Si	28	0.922297	0.046832	0.030872	
S	32	0.9493	0.0076	0.0429	0.0002

### Guidelines for optimal labelling measurement

**Figure 8:**
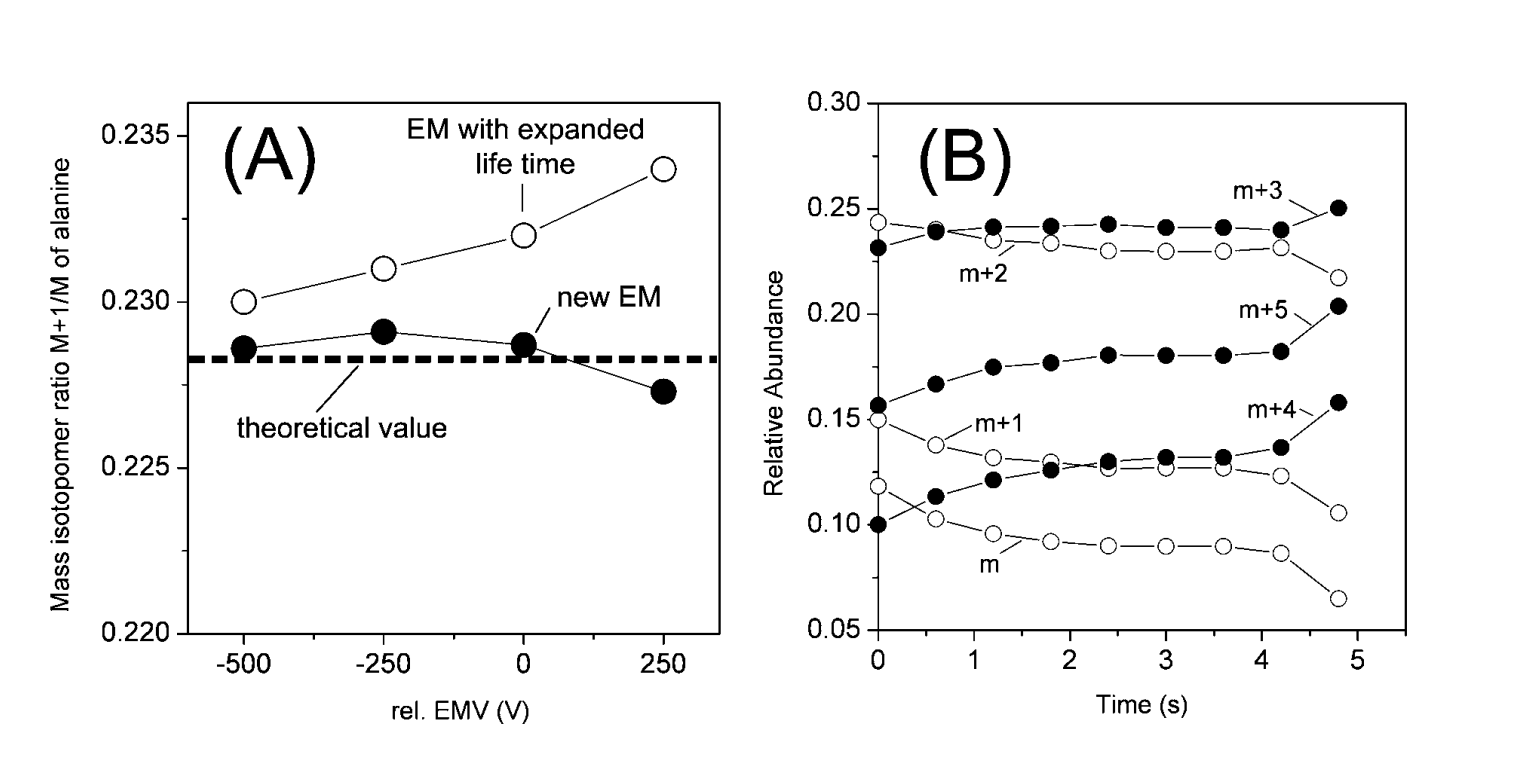
Influence of the life-time of the electron multiplier (A) and of isotope discrimination effects during the GC separation (B) on GC-MS labelling analysis. The effect of the electron multiplier is exemplified for the ratio between the single and the non labelled mass isotopomer fraction of naturally labelled TBDMS_2_-alanine whereby the dashed line represents the theoretical value and the experimental values result from measurement using an electron multiplier with extended life time and a new electron multiplier, respectively. The isotope discrimination effects are given for the different mass isotopomers of TBDMS_3_-glutamate.

Quantitative accuracy of the measurement is crucial for mass isotopomer analysis. Several sources of error may potentially affect the measurement result and should therefore be taken into account. First the signals have to be checked for purity, i.e. that isobaric interference of the analytes with co-eluting compounds does not occur. For the consistency check of GC-MS mass isotopomer distributions several efficient software tools have been developed [25,76,77]. A check can also be done experimentally by an additional cultivation on naturally labelled substrate in parallel to the tracer experiment and comparison of the mass isotopomer distributions from this study with theoretically expected values, calculated from the natural abundance of isotopes or experimental values from pure standard compounds. Another potential cause of inaccuracy is incomplete resolution of adjacent ions due to ion scattering and peak tailing. Therefore the mass spectrometer (lens system, quadrupole pre-filter, quadrupole rods, detector) has to be tuned to enable optimum mass-resolving capacity [50]. To obtain optimal signal intensity and avoid interference the signal for each mass isotopomer should be collected in SIM mode from the maximum of each mass peak. The *m/z *value at the peak maximum typically deviates from an integer value due to underlying atom masses contained in the ion. Nonlinearity of the detector response at different mass isotopomer ratios or different analyte amounts may further lead to false results [78]. Whereas such effects are observed for fatty acid methyl esters, mass isotopomer ratios of naturally labelled TBDMS-amino acids were not affected over a concentration range of 3 orders of magnitude [50]. Interference with background noise leads to false results for low abundance signals which therefore should not be considered for flux calculation. The same holds for signals above the saturation of the detector which underestimate the relative abundance of the most prominent mass isotopomer fractions. Such effects can be visualized by an inhomogeneous peak composition [50]. Reduction of the electron multiplier voltage (EMV) at the detector or sample dilution may compensate for such effects. One should further consider that the efficiency of electron multipliers can significantly decrease with life time. This can lead to a drift of the measured labelling pattern, i.e. mass isotopomer ratio, with increasing EMV or sample amount (Figure 8A). In such cases the multiplier should be changed. The high separation capacity of GC leads to isotope discrimination, as shown by the elution behaviour of the different mass isotopomers of TBDMS_3_-glutamate (Figure 8B). To adequately consider all mass isotopomers of a compound and obtain meaningful results, the average mass spectrum of the whole peak range has to be considered. This leads to a significantly reduced signal-to-noise ratio that excludes the use of labelling data from low abundance fragments [50].

## Experimental design, modelling and flux calculation

Metabolic flux analysis with GC-MS is usually performed as stationary flux analysis, whereby the metabolism is in metabolic and isotopic steady-state. Application examples are chemostat cultures [41] or batch cultures with balanced growth [4,23,66]. Additionally novel approaches have been developed to study fluxes also under non-steady-state conditions [79].

### Network topology

Generally, metabolic flux analysis by isotope labelling experiments requires a known topology of the biochemical network. Topological phenomena, such as metabolite channelling, compartmentation or zonation, mainly observed in eukaryotic systems, may influence the fate and the labelling pattern of metabolites in the network and must be considered, when interpreting obtained labelling data [80]. Metabolite channelling can prevent label scrambling of symmetric intermediates through orientation-conserved biocatalytic reactions as found for mitochondrial TCA cycle enzymes in mammalian cells [81] and yeast [82] and for pentose phosphate pathway (PPP) enzymes in yeast [83]. The compartments in eukaryotic cells lead to spatially separated metabolite pools and reactions, which has significant impact on labelling patterns. As example amino acid metabolism in yeast may occur in the cytosol, the mitochondrium or in both compartments, whereby the pathway location may vary between different species [84,85] or with cultivation conditions [18]. In tissue cultures zonation of metabolic activities may lead to heterogeneous labelling patterns as described for triose phosphate pools in rat liver [86]. The significant impact of metabolite channelling, compartmentation or zonation on labelling patterns, requesting their consideration in flux analysis, can be exploited by using isotope labelling studies to unravel these important features in biological systems [87]. Evidence for isotope effects on rate and equilibrium of metabolic reactions has not been gained, so that it appears justified to neglect them [16].

**Figure 9:**
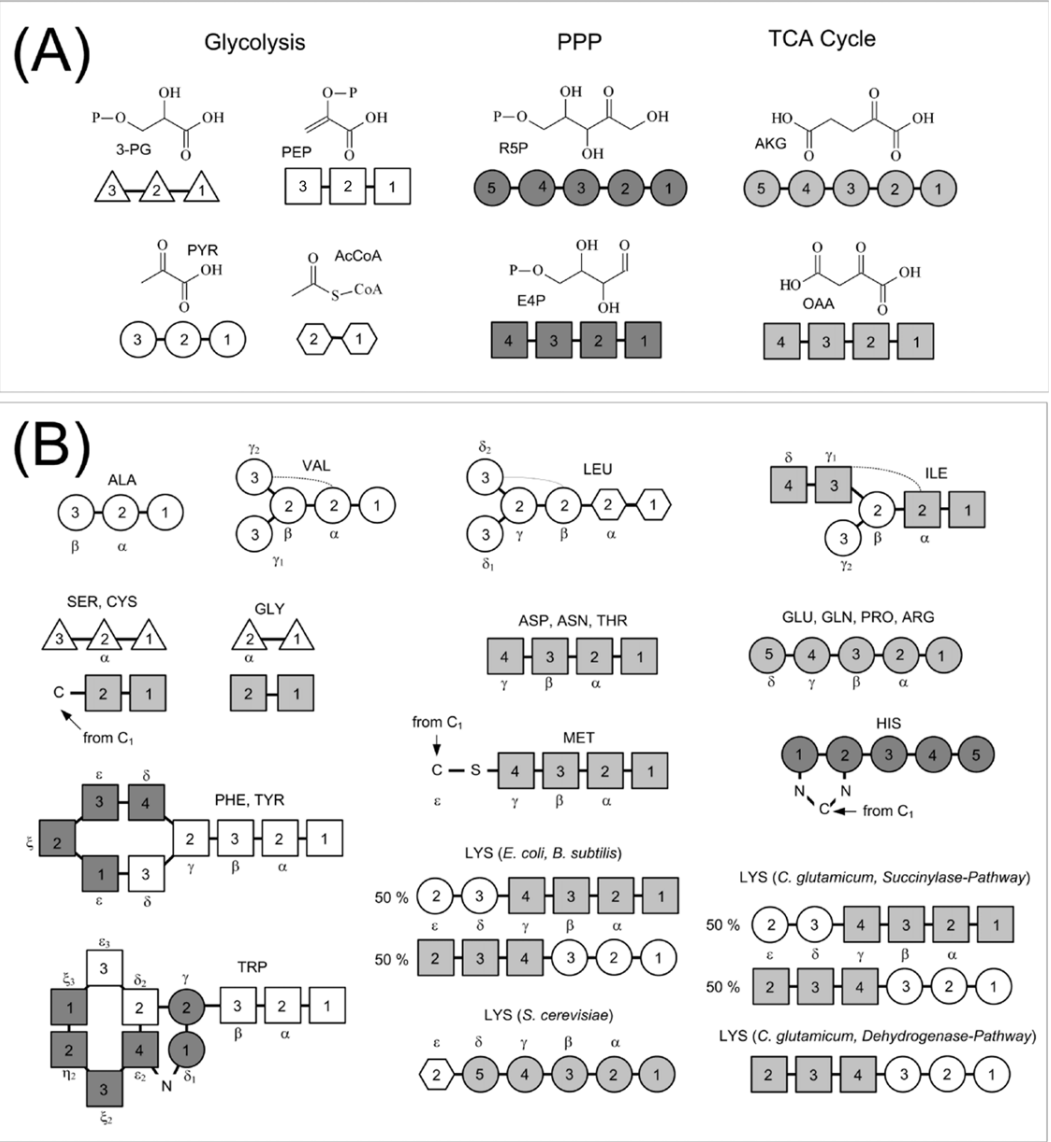
Relationship between the carbon skeleton of amino acids and the carbon skeleton of their metabolic precursors for the anabolic pathways in *E. coli*, *S. cerevisiae*, *C. glutamicum *and *B. subtilis*. The data are partly taken from [130].

The majority of GC-MS based flux approaches utilizes amino acid labelling patterns [16,50,85,88-90]. Knowing the precursor-amino acid relationships it is easy to deduce the labelling patterns of the precursor metabolites from labelling patterns of the amino acids. The relationship between precursor compounds from glycolysis, pentose phosphate pathway and TCA cycle and the amino acids for *E. coli*, *C. glutamicum*, *B. subtilis*, *P. putida *and *S. cerevisiae *is given in Figure 9. Except for lysine the synthesis of all amino acids is identical in all these microorganisms [91]. In *E. coli, B. subtilis *and *P. putida *one lysine biosynthetic route is available each from oxaloacetate and pyruvate, respectively, which includes a symmetric intermediate that results in label scrambling. Bakers's yeast utilizes a-ketoglutarate and acetyl-CoA as lysine precursors. *C. glutamicum *possesses two alternative pathways for lysine biosynthesis, whereby the in vivo activity of the two branches can vary with cultivation conditions or genetic modifications [92]. One should note that serine and glycine may not exclusively originate from their well known precursor 3-phosphoglycerate, but can also be formed from oxaloacetate via the threonine pathway and threonine aldolase [4,66].

### Choice of tracer substrates

The resolution of intracellular fluxes is strongly dependent on the ^13^C-labeling strategy. In GC-MS based approaches the use of [1-^13^C] glucose as well as the use of a mixture of [^13^C_6_] glucose and unlabeled glucose has proven useful to resolve important fluxes throughout the central metabolism in different organisms. Concerning the central metabolic pathways, a mixture of [^13^C_6_] glucose and unlabeled glucose is particularly useful to resolve fluxes downstream of PEP and some exchange fluxes that result in C-C bond cleavages, whereas the use of [1-^13^C] glucose is valuable for resolving the upper part of metabolism, in particular the oxidative PP pathway, glycolysis and the Entner-Doudoroff pathway [24]. Combining [U-^13^C] glucose and [1-^13^C] glucose, either in two separate experiments [19,21,24] or as a substrate mixture [20] may lead to an even better resolution of fluxes in the network. The resolution of a particular flux, however, depends on the network topology itself so general guidelines cannot be given. Questions concerning the determinability and the predicted accuracy of a certain flux together with an optimal experimental approach can be effectively answered by computer based experimental design [64,70,93]. One should note that the flux calculation is rather sensitive to the mixing ratio of two differently labelled compounds applied at the same time, e.g. [^13^C_6_] glucose and naturally labelled glucose. This mixing ratio should be exactly known, considering the facts that during storage some labelled compounds are hygroscopic. For glucose this can be taken into account by 8 h drying at 80°C prior to weighting.

### Flux calculation, statistics and goodness of fit

The flux parameters are usually estimated from the ^13^C tracer studies data by minimization of the deviation between experimental and simulated labelling data using numerical routines [15] Hereby, the free flux parameters of interest, such as flux partitioning ratios or reversibilities are varied by the optimization function starting from initial values until an acceptable agreement between experimental and simulated labelling patterns is achieved. Different optimization functions such as gradient or adaptive random search functions are available. To increase the probability of the identification of a global optimum for the solution, the convergence is usually tested for different initial guesses. As error criterion a weighted sum of least squares (SLS) is typically used (Equation 2).

SLS=∑i(ri,exp⁡−ri,calcri,exp⁡)2sr,i2     (Eq. 2)
 MathType@MTEF@5@5@+=feaafiart1ev1aaatCvAUfKttLearuWrP9MDH5MBPbIqV92AaeXatLxBI9gBaebbnrfifHhDYfgasaacH8akY=wiFfYdH8Gipec8Eeeu0xXdbba9frFj0=OqFfea0dXdd9vqai=hGuQ8kuc9pgc9s8qqaq=dirpe0xb9q8qiLsFr0=vr0=vr0dc8meaabaqaciaacaGaaeqabaqabeGadaaakeaacqWGtbWucqWGmbatcqWGtbWucqGH9aqpdaaeqbqaamaalaaabaWaaeWaaeaadaWcaaqaaiabdkhaYnaaBaaaleaacqWGPbqAcqGGSaalcyGGLbqzcqGG4baEcqGGWbaCaeqaaOGaeyOeI0IaemOCai3aaSbaaSqaaiabdMgaPjabcYcaSiabdogaJjabdggaHjabdYgaSjabdogaJbqabaaakeaacqWGYbGCdaWgaaWcbaGaemyAaKMaeiilaWIagiyzauMaeiiEaGNaeiiCaahabeaaaaaakiaawIcacaGLPaaadaahaaWcbeqaaiabikdaYaaaaOqaaiabdohaZnaaDaaaleaacqWGYbGCcqGGSaalcqWGPbqAaeaacqaIYaGmaaaaaaqaaiabdMgaPbqab0GaeyyeIuoakiaaxMaacaWLjaWaaeWaaeaacqqGfbqrcqqGXbqCcqGGUaGlcqqGGaaicqaIYaGmaiaawIcacaGLPaaaaaa@5FD3@

**Figure 10:**
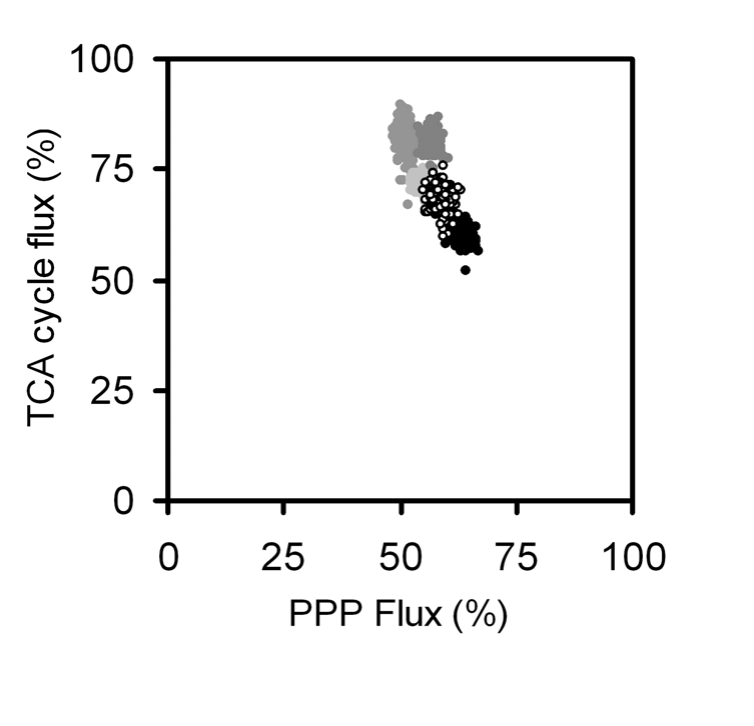
Statistical analysis of metabolic fluxes using a Monte-Carlo approach exemplified for flux through major NADPH generating pathways, the pentose phosphate pathway (PPP) and the TCA cycle. The calculation is based on a previous flux study of different lysine producing strains of *C. glutamicum *[21] and represents 250 independent flux estimations with statistically varied experimental data for each of the five strains shown.

By this, the differences between experimental (r_exp_) and calculated (r_calc_) labelling data, e. g. molar fractions of mass isotopomers ratios, are normalized and the resulting relative experimental errors of the corresponding MS measurements (s_r,i_) are used for weighting. As a result, data with relatively small error contribute to a higher extent, whereas the influence of data with a relatively high error and uncertainty has only a minor influence on the overall optimization result. Statistical analysis is of great importance in order to identify whether differences observed for intracellular flux distributions between different experiments can be really attributed to strain or condition specific differences. Such analysis can be performed by a Monte-Carlo approach including multiple parameter estimation runs with statistical variation of the experimental data [94,95]. The statistical variation is done such that random errors are added to the data sets, assuming a normal distribution of measurement errors around previously obtained mean values. Subsequently, multiple parameter estimations are carried out for each scenario, yielding multiple flux distributions with a corresponding mean value and a standard deviation for each intracellular flux parameter, from which confidence limits for the single parameters can be calculated. The high accuracy of intracellular fluxes determined by GC-MS based flux analysis is illustrated by a phase plane plot for different mutant strains of *C. glutamicum *(Figure 10). Based on previous experimental data [21] 250 independent estimates for the PPP and the TCA cycle flux with statistically varied experimental data for each strain show that, despite the strains differ only gradually in the corresponding fluxes, clear differentiation is possible. This is a great advantage as compared to NMR based flux approaches resulting in much higher uncertainty of intracellular fluxes [21]. Flux analysis using GC-MS allows a high goodness of fit, if all critical steps of the comprehensive approach are carefully addressed [19,21,22]. High deviation in selected mass isotopomer distributions leaves the estimated fluxes questionable. Such deviations may point at underlying errors in the performed study. Potential sources of errors include the labelling measurement itself, assumptions in the network topology, e.g. on available pathways, or carbon transfer in the underlying reactions, or simply errors in the complex metabolic model. Simulated and experimental labelling data as well as statistics on the calculated fluxes display important information that should always be included in the presentation of flux studies.

## Metabolic flux studies using GC-MS

The following chapter shows the application of GC-MS to metabolic flux analysis by different examples and should illustrate the broad application potential of such approaches in different fields of research. Whereas some of the examples focus on flux quantification of only a single or a few selected reactions, others aim at the analysis of larger parts of the metabolism.

### Bacteria, yeasts and fungi

GC-MS based metabolic flux analysis has been applied to various microorganisms. This includes several flux studies of *E. coli*, one of the most prominent model organisms. As example, different mutants were studied at miniaturized scale providing information on gene function [23]. Metabolic flux analysis utilizing GC-MS was used also to investigate the adaptation of *E. coli *to the loss of key metabolic enzymes [96], the function of soluble and membrane bound transhydrogenase [97] or growth on different substrates [26]. Additionally, the effect of single gene deletion on metabolic fluxes has been studied applying a combination of GC-MS and NMR for labelling analysis [26,98-102]. A number of studies have been carried out for the industrial amino acid producer *C. glutamicum*, providing important knowledge for rational strain improvement. This microorganism has been investigated with respect to the influence of different substrates in batch culture [19,22], fluxes in chemostat culture under substrate limitation [41], the comparison of different lysine producing mutants [4,21,103] and different phases of a production process [20]. Further prokaryotes studied on the level of intracellular carbon flux using GC-MS based approaches are *Bacillus subtilis *[79,104], *Bacillus clausii *[105]*or Streptomyces noursei *[106]. In another study flux ratio analysis was applied to compare fluxes in *Pseudomonas putida*, *Zymononas mobilis*, *Sinorhizobium meliloti*, *Rhodobacter sphaeroides *and *Lactococcus versutus *[107]. Among yeasts studied on the level of intracellular fluxes by GC-MS are *Saccharomyces cerevisiae *with studies on the influence of growth rate [18,27], or the comparison of different mutants [66], several members of the genus *Pichia *[84,88,108], *Phaffia rhodozyma *[28] or different other species [109]. Flux studies with fungi comprise e. g. biotechnologically relevant species such as *Penicillium chrysogenum *[29], *Aspergillus nidulans *[65] or *Aspergillus niger *[95].

### Mammalian cell cultures, tissues, animals and humans

Isotopic tracer experiments with GC-MS labelling analysis are utilized in the biomedical area field since many years [12,13]. Flux studies in this field significantly contributed to our current understanding on metabolic function in mammalian cell lines or organs or on the underlying metabolism related to diseases [110]. The inherent complexity of the networks with different compartments or tissue zones, metabolite channelling and complex dilution effects of the labelling often restricts the determination by a tracer experiment to selected flux parameters [15]. Flux studies of mammalian cell cultures comprise e.g. the comparison of different cell lines [30], the influence of butyrate on differentiation of adenocarcinoma cells [55], or the response of fasted rat hepatocytes to different substrates [31]. Extensive work has been carried out on quantifying fluxes in the central metabolism of tissue cultures. This involves studies on the pentose phosphate pathway in human hepatoma cells [111], or on anaplerosis, cataplerosis and the TCA cycle in perfused rat liver [35,36,112], and rat heart [32,113,114]. Another important application of stable isotopes and GC-MS is the in vivo quantification of polymer biosynthesis as exemplified by studies on DNA synthesis in rats [51,115,116], protein turn-over in humans [117] or lipidogenesis in humans [118]. Hereby, a mathematical approach is applied that allows the calculation of the labelling of the actual precursor molecules of a particular polymer [119-121].

## Outlook

Due to high robustness, sensitivity and versatility, GC-MS based approaches will play a central role as routine technology for future analysis of metabolic fluxes in various biological systems. The use of stable isotopes in combination with GC-MS for metabolic flux analysis will establish as important method in actual fields such as functional genomics, systems biology, and pharmaceutical research for drug development [122,123]. The compatibility with miniaturized cultivation tools will hereby allow the application to broad sets of mutants or cultivation conditions [24,103,124]. In addition to GC-MS also other mass spectrometry techniques, recently coming into focus, will be important tools for flux analysis. This involves LC-MS with excellent characteristics for dynamic flux measurements via labelling patterns of intracellular metabolites [71,125-127] or MALDI-TOF MS with ultra-fast measurement at low experimental effort [128] providing an outstanding innovation for high-throughput flux analysis e.g. of mutant libraries or culture conditions [124].
